# The predictive prognostic factors for polymyositis/dermatomyositis-associated interstitial lung disease

**DOI:** 10.1186/s13075-017-1506-7

**Published:** 2018-01-11

**Authors:** Yumiko Sugiyama, Ryusuke Yoshimi, Maasa Tamura, Mitsuhiro Takeno, Yosuke Kunishita, Daiga Kishimoto, Yuji Yoshioka, Kouji Kobayashi, Kaoru Takase-Minegishi, Toshiyuki Watanabe, Naoki Hamada, Hideto Nagai, Naomi Tsuchida, Yutaro Soejima, Hiroto Nakano, Reikou Kamiyama, Takeaki Uehara, Yohei Kirino, Akiko Sekiguchi, Atsushi Ihata, Shigeru Ohno, Shouhei Nagaoka, Hideaki Nakajima

**Affiliations:** 1 Department of Stem Cell and Immune Regulation, Yokohama City University Graduate School of Medicine, 3-9 Fukuura, Kanazawa-ku, Yokohama, 236-0004 Japan; 2 Department of Allergy and Rheumatology, Nippon Medical School Graduate School of Medicine, Tokyo, Japan; 3 Yokohama Minami Kyosai Hospital, Yokohama, Japan; 4 Center for Rheumatic Diseases, Yokohama City University Medical Center, Yokohama, Japan; 5 National Hospital Organization Yokohama Medical Center, Yokohama, Japan; 6 Chigasaki Municipal Hospital, Chigasaki, Japan; 7 Fujisawa City Hospital, Fujisawa, Japan

**Keywords:** Dermatomyositis, Polymyositis, Interstitial lung disease

## Abstract

**Background:**

Interstitial lung disease (ILD) is the principal cause of death in polymyositis/dermatomyositis (PM/DM). Here we investigated prognostic factors for death and serious infection in PM/DM-ILD using the multicenter database.

**Methods:**

We retrospectively reviewed baseline demographic, clinical and laboratory findings, treatment regimens and outcomes in patients with PM/DM-ILD. The distribution of ILD lesions was evaluated in four divided lung zones of high-resolution computed tomography images.

**Results:**

Of 116 patients with PM/DM-ILD, 14 died within 6 months from the diagnosis. As independent risk factors for early death, extended ILD lesions in upper lung fields (odds ratio (OR) 8.01, *p* = 0.016) and hypocapnia (OR 6.85, *p* = 0.038) were identified. Serious infection was found in 38 patients, including 11 patients who died of respiratory or multiple infections. The independent risk factors were high serum KL-6 (OR 3.68, *p* = 0.027), high initial dose of prednisolone (PSL) (OR 4.18, *p* = 0.013), and combination immunosuppressive therapies (OR 5.51, *p* < 0.001).

**Conclusion:**

The present study shows the progression of ILD at baseline is the most critical for survival and that infection, especially respiratory infection, is an additive prognostic factor under the potent immunosuppressive treatment.

**Electronic supplementary material:**

The online version of this article (doi:10.1186/s13075-017-1506-7) contains supplementary material, which is available to authorized users.

## Background

Polymyositis (PM) and dermatomyositis (DM) are idiopathic inflammatory myopathies (IIM) of unknown causes, which are often associated with extramuscular manifestations such as interstitial lung disease (ILD), arthropathy, cardiomyopathy, and malignancies. PM/DM-associated ILD (PM/DM-ILD) is a major cause of death [1, 2], with an estimated excess mortality rate of around 40%, and has a widely varied clinical course.

ILD is categorized according to histopathological findings into usual interstitial pneumonia, non-specific interstitial pneumonia (NSIP), organizing pneumonia, diffuse alveolar damage, respiratory bronchiolitis, desquamative interstitial pneumonia, and lymphoid interstitial pneumonia. In clinical practice, a multidisciplinary approach based on pathology findings, clinical symptoms, laboratory tests, imaging examinations, and respiratory function tests, is the gold standard for clinical diagnosis and classification of ILD [3]. High-resolution computed tomography (HRCT) scan is one of the most informative examinations for ILD. However, in comparison with idiopathic ILD, extrapulmonary findings including serological data are particularly important for making the diagnosis and determining the prognosis of connective tissue disease (CTD)-ILD including PM/DM-ILD. Indeed, previous studies have identified extrapulmonary findings as prognostic factors for PM/DM-ILD, such as old age [4], skin ulcer, ILD with low serum creatine kinase (CK) [5], non-Caucasian race, male sex [6], and seropositivity for anti-melanoma differentiation-associated protein 5 (MDA5) antibody (Ab) [7, 8]. It is important to optimize disease management based on the prognostic factors to improve clinical outcome.

In this study, we retrospectively assessed clinical data, treatments, and clinical outcomes in patients with PM/DM using the multicenter database to identify predictive prognostic factors for PM/DM-ILD.

## Methods

### Patient selection

One hundred sixteen patients with PM/DM-ILD, who had received initial treatment in six hospitals including Yokohama City University and the affiliated hospitals, from 2003 to 2016, were enrolled. The diagnosis of PM/DM was based on the Bohan and Peter criteria [9]. The modified Sontheimer criteria were adopted for the classification of clinically amyopathic dermatomyositis (CADM) [10]. Briefly, patients was categorized as having CADM if they presented with DM-specific skin disease but subclinical or no clinical evidence of proximal muscle weakness and myositis on laboratory, electrophysiologic, and/or radiologic evaluation more than 6 months after rash onset. This study was conducted in accordance with the Declaration of Helsinki, and informed consent was obtained from the patients and healthy controls. The study design was approved by the ethics committee of Yokohama City University.

### Collection of clinical data

We retrospectively obtained the data from the clinical charts of the individual patients. According to PM/DM-related manifestations such as presence of muscle weakness, typical dermatologic manifestations such as Gottron’s signs, Gottron’s papules, heliotrope rashes, and mechanic hands, fever, dysphasia, ILD, and malignancies, the patients were categorized into three groups according to the Bohan and Peter criteria and the modified Sontheimer criteria [9, 10]: PM; clinically amyopathic dermatomyositis (CADM); and classical dermatomyositis (DM). In addition, the diagnosis of cancer-associated myositis (CAM) was made in patients who had the complication of malignancy within 3 years before or after the onset of PM/DM.

We reviewed the disease onset, besides demographic findings including age, sex, habitual history such as smoking, and clinical findings such as laboratory data and imaging findings. The laboratory data included the routine biochemical and haematological data, KL-6, ferritin, those on blood gas analysis, and myositis-associated autoantibodies including anti-nuclear antibodies (ANA), anti-histidyl-tRNA synthetase (Jo-1), anti-aminoacyl transfer RNA synthetase (ARS), anti-MDA5, anti-transcriptional intermediary factor 1-γ (TIF1-γ), and anti- Sjögren’s syndrome A (SS-A) antibodies (Abs).

We also analyzed therapy and major clinical outcomes including survival, comorbidities, and complications. Common remission induction therapies for PM/DM-ILD were as follows; oral prednisolone (PSL) at a dose of 0.2–1.5 mg/kg of body weight per day, calcineurin inhibitors including tacrolimus or cyclosporine A, and administration of intravenous cyclophosphamide (IVCY), intravenous methyl-prednisolone (mPSL) pulse, and intravenous immunoglobulin (IVIg). All of the therapeutic strategies were determined by the attending physicians on the basis of comprehensive assessment of clinical manifestations and laboratory data. Serious infections were defined as conditions requiring hospitalization or extension of hospital stay for additional therapy with antimicrobial agents except for prophylactic purposes.

### HRCT

Presence of PM/DM-ILD was determined by chest HRCT findings such as ground glass opacity, reticular opacity, areas of consolidation, honeycombing, traction bronchiectasia, and linear opacity [11, 12]. Patients who needed initiation or intensification of immunosuppressive therapy for the lung lesions were included in the study. Distribution of ILD lesions was assessed in four lung zones, zone A (above the aortic arch), zone B (between the aortic arch and the level of the carina), zone C (between the level of the carina and the level of inferior pulmonary veins) and zone D (below the inferior pulmonary veins), according to a previous report [13]. In each zone, the extent of ILD lesions was semiquantitatively scored based on the percentage of the lung parenchyma involved: no involvement (0 points), 1–4% (1 point), 5–14% (2 points), 15–29% (3 points), 30–49% (4 points), and ≥ 50% involved (5 points). A total score was calculated by summing the individual area scores in each zone.

The HRCT images were independently reviewed by two rheumatologists well-trained in interpretation of chest CT images and blinded to the patients’ baseline data and treatment regimen. In the reliability assessment, the total score of the inter-evaluator and intra-evaluator coefficients of variation were good (κ = 0.71) and excellent (κ = 0.83), respectively. The inter-evaluator coefficient of variation in zone A to zone C was excellent (κ = 0.85, 0.84 and 0.92, respectively) and was good in zone D (κ = 0.72). The intra-evaluator coefficients of variation was excellent in every zone (κ = 0.84, 0.93, 0.93, and 0.80, respectively).

### Statistical analysis

To identify risk factors for death or complications from serious infections, statistical analysis was performed using SPSS software (IBM) and GraphPad Prism (GraphPad Software). We used the chi-square test or Fisher’s exact test for categorical variables, Student’s *t* test or analysis of variance for parametric analysis of continuous variables, or the two-tailed Mann-Whitney U test, Wilcoxon rank sum test, or Kruskal-Wallis test for non-parametric analysis. Receiver operating characteristic (ROC) analysis was performed to identify the appropriate cutoff values for variables that were significantly different on univariate analysis. We divided all the patients into two groups by the cutoff values and evaluated the survival curves statistically using the log-rank test. Cox proportional hazards regression and binomial logistic regression were performed as multivariate analysis to identify independent risk factors for death and serious infections. A *p* value < 0.05 was considered significant in all analyses.

## Results

### Patient characteristics and causes of death

This study enrolled 116 patients with PM/DM-ILD. Demographic, clinical, and laboratory data at diagnosis of PM/DM-ILD, major clinical outcomes including survival, complication with serious infection, and malignancy are shown in Table 1 and Additional file 1. The mean age was 56.0 ± 14.8 years and 83 (71.6%) were female. During the observation period, 28 patients (24.1%) died at 26.3 ± 34.9 months from diagnosis. It is of note that ILD was directly related to early death in 12 (85.7%) of 14 patients who died within 6 months after the diagnosis.

**Table 1:** The demographic data on patients with PM/DM with ILD (excerption^a^)

Variables (n = 116 patients)		Values
Women (*n* (%))		83/116 (71.6%)
Age (years)		56.0 ± 14.8^b^
Type (*n*)		PM 22, DM 51, CADM 43
Baseline data	CK (U/l)	360 (115–1496)^c^
	LDH (U/l)	369 (279–526)^c^
	KL-6 (U/ml)	673 (453–1030)^c^
	CRP (mg/dl)	0.57 (0.15–1.78)^c^
	Lymphocytes (/μl)	971 (696–1386)^c^
	Albumin (g/dl)	3.42 ± 0.56^b^
	PaCO_2_ (mmHg)	37.3 (34.3–40.3)^c^
	Ferritin (ng/ml)	360 (165–843)^c^
Autoantibody (*n* (%))	Anti-Jo-1 Ab	21/114 (18.4%)
	Anti-ARS Ab	9/45 (20.0%)
	Anti-MDA5 Ab	8/31 (25.8%)
	Anti-TIF-1γ Ab	2/2 (100%)
Malignancy (<3 years) (*n* (*%*))		21/112 (18.8%)
Treatment (*n* (%))	Initial PSL dose (mg/kg/day)	0.83 ± 0.29^b^
	mPSL pulse	77/116 (66.4%)
	IVCY	48/116 (41.4%)
	Calcineurin inhibitor	81/115 (70.4%)
	Combination therapy^d^	40/116 (34.5%)
Prognosis (*n* (%))	Death	28/116 (24.1%)
	Serious infection	38/116 (32.8%)

*CK* creatine kinase, *LDH* lactate dehyrdrogenase, CRP C-reactive protein, *PaCO*_*2*_ arterial partial pressure of carbon dioxide, *PSL* prednisolone, *mPSL* intravenous methyl-prednisolone, *IVCY* intravenous cyclophosphamide, *PM* polymyositis, *DM* dermatomyositis, *CADM* clinically amyopathic dermatomyositis

^a^See also Additional file 1: Table S1

^b^The data are shown as the mean ± standard deviation

^c^Values are the median (interquartile range)

^d^Combination therapy includes glucocorticoid, IVCY and calcineurin inhibitors

**Fig. 1:**
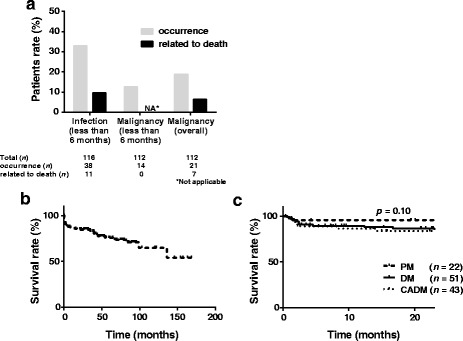
Survival curves for patients with polymyositis/dermatomyositis-interstitial lung disease (PM/DM-ILD) over the observation time. **a** Frequency of infections and malignancies in patients with PM/DM-ILD up to 6 months after initiating immunosuppressive therapy and within three years before and after diagnosis of PM/DM in the overall observation period (gray columns). Frequency of the patients with PM/DM who died from infection and malignancy within 6 months and in the overall observation period (black columns) are also shown. **b** The survival curve for the patients with PM/DM-ILD. It shows that there are two phases with a high rate of death. **c** The survival curves for each PM/DM subtype reveals no significant difference among the three subtypes. *CADM* clinically amyopathic dermatomyositis

Serious infections were complications in 38 (32.8%) of all the studied patients with PM/DM-ILD within the first 6 months after the diagnosis (Fig. 1a and Table 1). They were also related to 11 (78.6%) of 14 early deaths, which corresponds to 9.5% of all the patients (Fig. 1a). Eight patients had both ILD exacerbation and complication of serious infections, each of which could be lethal. Thus, it was hard to determine which was primarily responsible for the deaths.

Complication with malignancy, which appeared within 3 years before and after the diagnosis of PM/DM-ILD, so-called cancer-associated myositis (CAM), was identified in 21 (18.8%) of all the studied patients, including 3 with PM (15.0%), 12 with DM (24.5%) and 6 with CADM (14.0%) (Fig. 1a and Table 1). In 16 of them, malignancy was identified within a year from the diagnosis of PM/DM-ILD. The primary lesions of malignancies were diverse, distributed in the breast, ovary, lung, thyroid gland, pancreas, gallbladder, and colorectum. All seven deaths from malignancy, which corresponds to 6.3% of all the patients with malignancy, occurred more than 6 months after the diagnosis of PM/DM-ILD (Fig. 1a). No patients died from malignancy within 6 months after the diagnosis.

These data indicate that ILD is a critical prognostic factor in patients with PM/DM-ILD and that infection additively contributes to unfavorable clinical outcomes. The survival curves indicate that there are two distinct waves of fatal events in patients with PM/DM-ILD (Fig. 1b). The first wave is generated by ILD and infection in the early phase, whereas the second one is due to complicated malignancy later.

### Comparison of clinical features among PM/DM subtypes in PM/DM-ILD

We diagnosed ILD based on HRCT findings in 116 patients, including 22 with PM (19.0%), 51 with DM (44.0%) and 43 with CADM (37.1%) (Table 2 and Additional file 2). All PM/DM subtypes were determined by manifestations that appeared throughout the clinical course.

**Table 2:** Comparison of demographic data among each PM/DM subtype in PM/DM-ILD patients (excerption^a^)

PM/DM-ILD (n = 116)		PM (n = 22)	DM (n = 51)	CADM (n = 43)	*P* value
Women (*n* (%))		13/22 (59.1%)	40/51 (78.4%)	30/43 (69.8%)	0.23
Age (years)		56.2 ± 14.9^b^	60.0 ± 13.3^b^	51.3 ± 15.4^b^	0.016**
Baseline data	CK (U/l)	1956 (1067–4399)^c^	609 (211–1773)^c^	83 (57–183)^c^	<0.001**
	LDH (U/l)	449 (374–564)^c^	437 (341–590)^c^	284 (242–343)^c^	<0.001**
	KL-6 (U/ml)	685 (490–1709)^c^	759 (463–1129)^c^	657 (413–911)^c^	0.35
	CRP (mg/dl)	0.71 (0.18–1.95)^c^	0.62 (0.16–1.94)^c^	0.49 (0.15–1.32)^c^	0.44
	Lymphocyte (/μl)	1346 (983–1553)^c^	971 (696–1386)^c^	825 (592–1041)^c^	0.022*
	Ferritin (ng/ml)	258 (165–305)^c^	960 (719–1365)^c^	361 (116–645)^c^	0.032*
Autoantibody (*n* (%))	Anti-Jo-1 Ab	8/21 (38.1%)	10/51 (19.6%)	3/42 (7.1%)	0.011*
	Anti-MDA5 Ab	0/1 (0%)	4/17 (23.5%)	4/13 (30.8%)	NA^e^
Malignancy (<3 years) *n* (*%*)		3/20 (15.0%)	12/49 (24.5%)	6/43 (14.0%)	0.39
HRCT	Zone A	1.0 (0–1.0)^c^	1.0 (0–1.0)^c^	1.0 (0–1.0)^c^	0.90
	Zone B	1.0 (1.0–1.8)^c^	1.0 (0–2.0)^c^	1.0 (0–1.0)^c^	0.58
	Zone C	2.0 (1.3–3.0)^c^	1.0 (1.0–3.0)^c^	1.0 (1.0–2.0)^c^	0.22
	Zone D	4.0 (3.0–4.8)^c^	3.0 (2.0–4.0)^c^	3.0 (2.0–3.0)^c^	0.033*
	Zone total	8.0 (5.3–10.8)^c^	6.0 (3.0–10.0)^c^	6.0 (4.0–7.0)^c^	0.25
Treatment (*n* (%))	Initial PSL dose (mg/kg/day)	0.77 ± 0.25^b^	0.92 ± 0.28^b^	0.72 ± 0.32^b^	0.004**
	mPSL pulse (*n* (%))	10/22 (45.5%)	35/51 (68.6%)	32/43 (74.4%)	0.059
	IVCY	6/22 (27.3%)	19/51 (37.3%)	23/43 (53.5%)	0.092
	Calcineurin inhibitor	8/22 (36.4%)	36/50 (72.0%)	37/43 (86.0%)	<0.001**
	Combination therapy^d^	2/22 (9.1%)	18/51 (35.3%)	20/43 (46.5%)	0.011*
Prognosis (*n* (%))	Death	6/22 (27.3%)	15/51 (29.4%)	7/43 (9.6%)	0.31

*PM* polymyositis, *DM* dermatomyositis, *ILD* interstitial lung disease, *HRCT* high-resolution computed tomography, *CK* creatine kinase, *LDH* lactate dehyrdrogenase, CRP C-reactive protein, *PaCO*_*2*_ arterial partial pressure of carbon dioxide, *PSL* prednisolone, *mPSL* intravenous methyl-prednisolone, *IVCY* intravenous cyclophosphamide, *CADM* clinically amyopathic dermatomyositis

^a^See also Additional file 2: Table S2

^b^The data are shown as the mean ± standard deviation

^c^Values are the median (interquartile range)

^d^Combination therapy includes glucocorticoid, IVCY and calcineurin inhibitors

^e^Not applicable

*p < 0.05, **p < 0.01

At baseline, the patients in the DM group were significantly older than those in the other two groups. Among the laboratory data, serum CK, LDH, and CRP were significantly lower in the CADM group than the other two groups, while serum ferritin level was highest in the DM group. The serological study revealed that anti-Jo-1 Ab was most prevalent in the PM group (38.1%) followed by the classical DM group (19.6%) and the CADM group (7.1%). Although anti-MDA5 Ab has been emphasized as a biomarker of CADM-associated ILD, the autoantibody was detected not only in the CADM (30.8%) but also in the classical DM group (23.5%), though it was examined only in some of the patients.

The distribution of ILD lesions assessed by the zone score was not different among the three groups except for the zone D score, which was the higher in the PM than in the other groups. The initial dose of PSL was significantly higher in the DM group than in the others, while the frequency of methyl PSL pulse therapy was comparable among the three groups (Table 2). On the other hand, remission induction therapy was more intensified by adding the concurrent use of calcineurin inhibitors and/or IVCY to corticosteroids in the CADM group as compared with the other groups; combination therapy was given in 47% of patients with CADM. This is probably in line with a number of previous studies that have shown that CADM is associated with unfavorable clinical outcomes [14]. However, our study showed no differences in the survival curves between the CADM and the classical DM groups (Fig. 1c). On the other hand, all but one of the patients with PM survived the observation period.

### Comparison of clinical features between survivors and non-survivors in PM/DM-ILD

To determine prognostic factors for early death within fewer than 6 months after the diagnosis of PM/DM-ILD, we next compared baseline clinical features between the survivors and the short-term non-survivors (Table 3 and Additional file 3). There were no significant differences between the periods from the initial manifestation to the initiating treatment. The short-term non-survivors among patients with PM/DM-ILD were characterized by having high serum KL-6, lymphopenia, hypoalbuminemia, hypocapnia, and extensive ILD.

**Table 3:** Comparison of demographic data between survivors and non-survivors of PM/DM-ILD patients (excerption^a^)

PM/DM-ILD (n = 116)		Non-survivor^e^ (n = 14)	Survivor (n = 102)	*P* value
Women (*n* (%))		8/14 (57.1%)	75/102 (73.5%)	0.22
Type (*n*)		PM 1, DM 8, CADM 5	PM 21, DM 43, CADM 38	0.41
Age (years)		60.1 ± 9.6^b^	55.5 ± 15.4^b^	0.14
Baseline data	KL-6 (U/ml)	935 (809–1230)^c^	612 (431–1023)^c^	0.016*
	Lymphocyte (/μl)	714 (398–909)^c^	1,013 (730–1425)^c^	0.002**
	Albumin (g/dl)	3.03 ± 0.54^b^	3.46 ± 0.55^b^	0.014*
	PaCO_2_ (mmHg)	32.6 (31.2–36.0)^c^	37.8 (35.3–40.7)^c^	0.005**
HRCT	Zone A	2.0 (1.0–2.0)^c^	1.0 (0–1.0)^c^	<0.001**
	Zone B	2.0 (1.0–2.8)^c^	1.0 (0–1.0)^c^	0.001**
	Zone C	2.0 (1.0–3.8)^c^	1.0 (1.0–2.0)^c^	0.069
	Zone D	4.0 (3.0–4.8)^c^	3.0 (2.0–4.0)^c^	0.021*
	Zone total	10.0 (6.5–13.5)^c^	6.0 (4.0–8.3)^c^	0.004**
Treatment (*n* (%))	Initial PSL dose (mg/kg/day)	0.96 ± 0.24^b^	0.80 ± 0.31^b^	0.061
	mPSL pulse (*n* (%))	14/14 (100%)	63/102 (61.8%)	0.002**
	IVCY (*n* (%))	11/14 (78.6%)	37/102 (36.3%)	0.003**
	Calcineurin inhibitor	13/14 (92.9%)	68/101 (67.3%)	0.062
	Combination therapy^d^	10/14 (71.4%)	30/102 (29.4%)	0.005**
Outcome (*n* (%))	Serious infection	11/14 (78.6%)	27/102 (26.5%)	<0.001**

*PM* polymyositis, *DM* dermatomyositis, *ILD* interstitial lung disease, *HRCT* high-resolution computed tomography, *CK* creatine kinase, *LDH* lactate dehyrdrogenase, CRP C-reactive protein, *PaCO*_*2*_ arterial partial pressure of carbon dioxide, *PSL* prednisolone, *mPSL* intravenous methyl-prednisolone, *IVCY* intravenous cyclophosphamide, *CADM* clinically amyopathic dermatomyositis

^a^See also Additional file 3: Table S3

^b^The data are shown as the mean ± standard deviation

^c^Values are the median (interquartile range)

^d^Combination therapy includes glucocorticoid, IVCY and calcineurin inhibitors

^e^The non-survivor group includes the patients who died within 6 months after the diagnosis of PM/DM

*p < 0.05, **p < 0.01

**Fig. 2:**
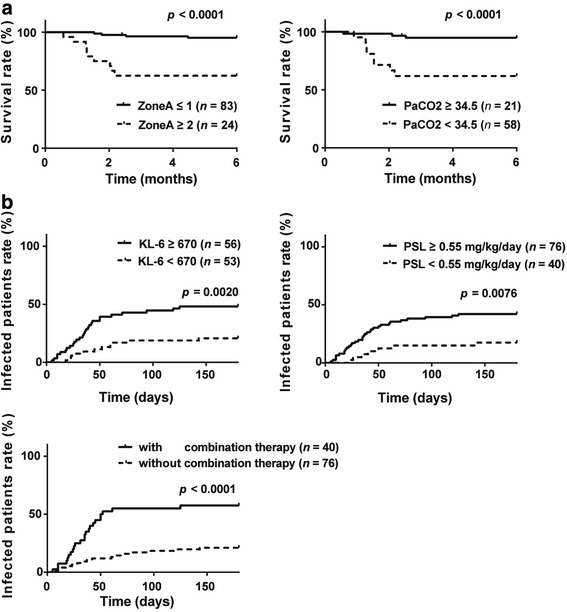
Survival curves and infection-free rates in patients with polymyositis/dermatomyositis-interstitial lung disease (PM/DM-ILD) 6 months after baseline. Patients are divided into two groups based on the cutoff points of individual variables, which were determined by receiver operating characteristic analysis. **a** high score in zone A and low arterial partial pressure of carbon dioxide (PaCO_2_) are significantly associated with early death. The cutoff point for zone A is 1.3 (between 1 and 2) and that for PaCO_2_ is 34.5 mmHg. **b** High serum KL-6, high initial prednisolone (PSL) dose and treatment with combination therapy are significantly associated with serious infections. The cutoff point for KL-6 is 670 U/ml and that for initial PSL dose is 0.55 mg/kg body weight per day

We determined the cutoff points for individual variables to separate survivors from non-survivors using ROC analysis, and conducted multivariate analysis using binomial logistic regression. We found that high zone A score (OR 8.01, *p* = 0.016) and low arterial partial pressure of carbon dioxide (PaCO_2_) (OR 6.85, *p* = 0.038) were independently associated with early death. ROC analysis revealed that the cutoff points for the zone A score was 1.3 (between 1 and 2) for distinguishing the short-term non-survivors from survivors. The survival curve for patients with a zone-A score > 1 was significantly inferior to those in the other patients at any time point (Fig. 2a). Likewise, there was a significant difference in the survival curves between patients with and without hypocapnia, < 34.5 mmHg of PaCO_2_ (Fig. 2a). Both of the risk factors were associated with progression of ILD, especially extension of ILD lesions into the upper lung fields, zone A, at baseline.

### Comparison of clinical features between patients with and without the complication of serious infections in PM/DM-ILD

As shown in Table 3, the complication of infection was another candidate for the critical prognostic factor especially for survival in the early phase. By multivariate analysis using binomial logistic regression, we identified serious infection as an independent factor for early death after starting treatment in PM/DM-ILD (OR 6.49, *p* = 0.012). Therefore, our analysis focused on serious infectious events within 6 months from starting treatment in PM/DM-ILD.

Bacterial infection was the most frequent among causative pathogens. A total of 34 bacterial infection events consisted of 16 cases of pneumonia, 9 of urinary tract infections, 4 of catheter-related bloodstream infections, 3 of cellulitides, one of lung abscess, and one of femoral intramuscular abscess. Fungal infection was identified in 15 patients including 9 with pneumocystis pneumonia, which was prevalent in patients not receiving prophylactic therapy with sulfamethoxazole/trimethoprim (ST) agents. As for viral infection, there were 23 patients with cytomegalovirus (CMV) antigenemia requiring antiviral therapy under regular monitoring (Additional file 3), although none of them developed serious organ involvement.

Multiple infections were identified in 13 patients, including 10 short-term non-survivors. Respiratory infection, which was the most common focus, was identified in 13 patients, including 10 short-term non-survivors. Moreover, early death was associated with multiple infections including respiratory infection, which was directly involved in lethal events.

Comparative analysis of the baseline variables between patients with and without infection revealed that patients in the infection group were more likely to have high serum LDH, KL-6, CRP and hypoalbuminemia, lymphopenia, hypocapnia, and high ILD score, especially in the upper lung fields (Table 4 and Additional file 4). Most of the risk factors were shared between death and serious infection. In addition, however, unlike survival prognostic factors, the univariate analyses extracted several therapy-related factors, including a high dose of initial PSL, mPSL pulse therapies, IVCY, calcineurin inhibitors and combination therapy, but not cumulative PSL dose, as risk factors for serious infections.

**Table 4:** Comparison of demographic data between PM/DM-ILD patients with or without complication of serious infection^a^ (excerption^b^)

PM/DM-ILD (n = 116)		Infection (n = 38)	Non-infection (n = 78)	*P* value
Women (*n* (%))		24/38 (63.2%)	59/78 (75.6%)	0.16
Type (*n*)		PM 7, DM 24, CADM 7	PM 15, DM 27, CADM 36	0.007**
Age (years)		58.9 ± 13.9^c^	54.7 ± 15.2^c^	0.15
Baseline data	LDH (U/l)	420 (335–574)^d^	343 (258–486)^d^	0.005**
	KL-6 (U/ml)	848 (605–1231)^d^	572 (405–978)^d^	0.014*
	CRP (mg/dl)	0.9 (0.34–1.99)^d^	0.30 (0.10–1.51)^d^	0.032*
	Lymphocyte (/μl)	848 (552–1177)^d^	1,008 (735–1435)^d^	0.040*
	Albumin (g/dl)	3.18 ± 0.52^c^	3.53 ± 0.55^c^	0.003**
	PaCO_2_ (mmHg)	36.0 (31.5–38.8)^d^	38.0 (35.5–41.5)^d^	0.016*
HRCT	Zone A	1.0 (0–2.0)^d^	1.0 (0–1.0)^d^	0.054
	Zone B	1.0 (1.0–2.0)^d^	1.0 (0–1.0)^d^	0.049*
	Zone C	2.0 (1.0–3.0)^d^	1.0 (1.0–2.0)^d^	0.036*
	Zone D	3.0 (2.0–4.0)^d^	3.0 (2.0–4.0)^d^	0.068
	Zone total	7.0 (4.0–11.0)^d^	6.0 (4.0–8.0)^d^	0.031*
Treatment (*n* (%))	Initial PSL dose (mg/kg/day)	0.94 ± 0.30^c^	0.76 ± 0.29^c^	0.005**
	mPSL pulse (*n* (%))	31/38 (81.6%)	46/78 (59.0%)	0.016*
	IVCY (*n* (%))	25/38 (65.8%)	23/78 (29.5%)	<0.001**
	Calcineurin inhibitor	34/38 (89.5%)	47/77 (61.0%)	0.002**
	Combination therapy^e^	23/38 (60.5%)	17/78 (21.8%)	<0.001**
Outcome (*n* (%))	Death	11/38 (28.9%)	3/78 (3.8%)	<0.001**

*PM* polymyositis, *DM* dermatomyositis, *ILD* interstitial lung disease, *HRCT* high-resolution computed tomography, *CK* creatine kinase, *LDH* lactate dehyrdrogenase, CRP C-reactive protein, *PaCO*_*2*_ arterial partial pressure of carbon dioxide, *PSL* prednisolone, *mPSL* intravenous methyl-prednisolone, *IVCY* intravenous cyclophosphamide, *CADM* clinically amyopathic dermatomyositis

^a^The infection group includes the patients with PM/DM-ILD who had the complication of serious infections, which needed intravenous antibiotic therapy or longer hospitalization within 6 months after diagnosis

^b^See also Additional file 4: Table S4

^c^The data are shown as the mean ± standard deviation

^d^Values are the median (interquartile range)

^e^Combination therapy includes glucocorticoid, IVCY and calcineurin inhibitors

*p < 0.05, **p < 0.01

After the cutoff points of individual numerical parameters were determined by ROC analysis, multivariate analysis using binomial logistic regression identified high serum KL-6 (OR 3.68,　*p* = 0.027), initial PSL dose (OR 4.18, *p* = 0.013), combination therapy (OR 5.51, *p* < 0.001) and male sex (OR 3.38, *p* = 0.024) as independent risk factors for serious infections. As shown in Fig. 2b, there were significant differences in the infection-free survival curves between high-risk and low-risk groups for individual independent risk factors except for sex.

## Discussion

Consistent with some previous reports [1, 2], we had identified the existence of ILD at baseline and the complication of serious infection as risk factors for death (OR 6.10, *p* = 0.010, and OR 2.46, *p* = 0.038, respectively) in patients with PM/DM (n = 188) in our multicenter database. In this study, we focused on the patients with PM/DM who had ILD and investigated their independent prognostic factors.

This study showed that presence of the ILD lesion in the upper lung fields and hypocapnia were independently associated with unfavorable clinical outcomes, especially early death in patients with PM/DM-ILD. In general, mortality in PM/DM-ILD has two waves [14–16]. Exacerbation of ILD and/or complication of serious infections are mainly responsible for the first peak [14, 17]. The present study revealed that hypocapnia and extent of ILD lesions up to the upper lung fields are the independent prognostic factors for PM/DM-ILD, indicating that progression and ILD severity was the most critical factor in the early phase. In concordance with the present study, we have previously shown that positron emission tomography (PET)-CT scan visualizes active ILD lesions and the extent is closely associated with progression of ILD in connective tissue diseases including PM/DM [18]. The second wave of mortality is flatter than the first one and is mainly due to malignancy in patients with CAM [14, 19].

There is accumulating evidence that patients with CADM and anti-MDA5 Ab frequently have acute progressive ILD requiring potent immunosuppressive therapy [20, 21]. However, neither CADM nor anti-MDA5 Ab was identified as prognostic factors in this study. The discrepancy from the previous studies was caused by the incomplete study of anti-MDA5 Ab, which was examined in some of the patients, mainly in those diagnosed more recently. Our study did not show any difference in survival prognosis between CADM and classical DM. The data suggest that serological anti-MDA5 Ab is more closely associated with acute progressive ILD than CADM as a clinical phenotype, as previous studies have shown that patients with classical DM anti-MDA5 Ab present with rapidly progressive ILD as do patients with seropositive CADM [22–24]. Serum ferritin level has been shown to be a biomarker for the activity of ILD in patients with PM/DM [25]. Unlike the previous studies, however, we did not identify a significant association between serum ferritin and ILD-related death. In this study, we only analyzed the baseline data but not those in those with the most active disease, presumably leading to the discrepancy.

There is no evidence-based treatment for PM/DM-ILD, because of lack of prospective randomized controlled trials. In the real world, the first-line therapy for PM/DM-ILD is high-dose glucocorticoid therapy with or without immunosuppressants [26–29]. As several lines of study suggest, potent immunosuppressive combination therapy using high-dose glucocorticoid, calcineurin inhibitors, and cyclophosphamide should be considered as induction therapy for acute progressive ILD, particularly in patients with CADM who are positive for anti-MDA5 Ab, because of unfavorable prognosis, as mentioned above [30].

On the other hand, it has been shown that infection is one of the major complications during treatment for PM/DM. Some recent studies showed that the serious infection occurred in 26–37% of patients with PM/DM during treatment [17, 31–33]. The incidence rate of serious infection in our database cohort was quite similar to those in these studies. The rate of serious infection as a cause of death was also similar to those of the previous studies (2–22%) [5, 17, 31, 33, 34].

Intensive immunosuppressive therapies are associated with high risk of infection [17, 31, 35]. Nzeusseu et al. also demonstrated that high dose of initial corticosteroids is associated with increased adverse events including infection but not with improving clinical outcomes of myopathies [36]. Another group identified different sets of risk factors including esophageal involvement, ventilator insufficiency, malignancy, myalgia, and use of methotrexate, but not a cumulative dose of glucocorticoids or use of immunosuppressants in patients with PM/DM [32]. However, these reports might be too old to assess recent styles of combination immunosuppressive therapies including calcineurin inhibitors. Unlike previous studies, besides therapeutic factors including the initial PSL dose, and combination immunosuppressive therapy, the present study showed high serum KL-6 to be a risk factor for infection, suggesting that progressive ILD predisposes to the local complication of infection.

We would like to emphasize that our study identified serious infections, especially with respiratory infection, as another prognostic factor for PM/DM-ILD patients. It is likely that complication with respiratory infection further accelerates the progression of respiratory insufficiency in a patient with PM/DM-ILD requiring intensive immunosuppressive therapies, resulting in a fatal event. Indeed, such clinical courses were observed in 10 of 14 patients with PM/DM-ILD who died in this study.

Our data suggest the reconsideration of the appropriate therapeutic regimen for PM/DM-ILD. The present retrospective observational analyses suggests that the clinical outcome is favorable in the regimen using 0.5 mg/kg body weight per day of PSL with a calcineurin inhibitor compared with that using 1.0 mg/kg/day of PSL or IVCY, although the choice of either regimen has not been controlled. This issue should be analyzed in a prospective study in the future. At this moment, we would like to emphasize that infection should be controlled by prophylaxis based on screening, and by early detection using monitoring and subsequent treatment, in the management of patients with PM/DM-ILD receiving intensive immunosuppressive therapies.

There are several limitations in this study. First, the previously reported prognostic factors for PM/DM-ILD, such as myositis-specific autoantibodies (MSA) and ferritin, were not collected comprehensively because of the retrospective study design and unavailability of stocked serum as discussed above. It can be attributed to the fact that measurement of MSA, except for anti-Jo-1 Ab, had not been commercially available and the relationship between serum ferritin level and mortality in PM/DM-ILD had not yet been identified in the significant part of the study period. Second, therapeutic decision-making relied on the individual attending physicians without use of consistent protocols.

## Conclusions

In conclusion, we found both local and extrapulmonary findings, including immunosuppressive therapies, were risk factors for fatal events and serious infections in patients with PM/DM-ILD, based on clinical findings.

## Additional files

Additional file 1: Table S1. The demographic data of patients with PM/DM with ILD. (PDF 37 kb)

Additional file 2: Table S2. Comparison of demographic data among each PM/DM subtype in PM/DM-ILD patients. (PDF 102 kb)

Additional file 3: Table S3. Comparison of demographic data between survivors and non-survivors of PM/DM-ILD patients. (PDF 92 kb)

Additional file 4: Table S4. Comparison of demographic data between PM/DM-ILD patients with or without complication of serious infection. (PDF 92 kb)

## Abbreviations

Ab: Antibody
ANA: Anti-nuclear antibodies
Anti-ARS Ab: Anti-aminoacyl transfer RNA synthetase antibody
Anti-MDA5 Ab: Anti-melanoma differentiation-associated protein 5 antibody
Anti-SS-A Ab: Anti-Sjögren’s syndrome A antibody
Anti-TIF1-γ Ab: Anti-transcriptional intermediary factor 1-γ antibody
CADM: Clinically amyopathic dermatomyositis
CAM: Cancer-associated myositis
CK: Creatine kinase
CMV: Cytomegalovirus
CT: Computed tomography
DM: Dermatomyositis
HRCT: High-resolution computed tomography
IIM: Idiopathic inflammatory myopathies
ILD: Interstitial lung disease
IVCY: Intravenous cyclophosphamide
IVIg: Intravenous immunoglobulin
Jo-1: anti-histidyl-tRNA synthetase
mPSL: Intravenous methyl-prednisolone
MSA: Myositis-specific autoantibodies
OR: Odds ratio
PaCO_2_: arterial partial pressure of carbon dioxide
PM: Polymyositis
PSL: Prednisolone
ROC: Receiver operating characteristic
