# Supplementary material for: The predictive prognostic factors for polymyositis/dermatomyositis-associated interstitial lung disease
Source: Arthritis Res Ther. 2018 Jan 11;20:7. doi: 10.1186/s13075-017-1506-7 (PMC5765702; doi:10.1186/s13075-017-1506-7)
Supplement: Supplementary file 1 — The demographic data of patients with PM/DM with ILD. (PDF 37 kb) [file 13075_2017_1506_MOESM1_ESM.pdf]

Table S1 The demographic data of PM/DM patients with ILD.

**PM/DM-ILD (*n* = 116)**		
Women *n* (%)		83 /116 (71.6%)
Age (year)		56.0 ± 14.8[a]
Smoking *n* (%)		37 /106 (34.9%)
Follow-up period (months)		47.0 [21.0-75.5] [b]
Type (*n*)		PM 22, DM 51, CADM 43
Manifestation *n* (%)	Eruption	97 /115 (84.3%)
	Muscle weakness	76/115 (66.1%)
	Dysfunction of swallowing	22/114 (19.3%)
	fever	53/115 (46.1%)
Baseline data	CK (U/l)	360 [115-1,496] [b]
	LDH (U/l)	369 [279-526] [b]
	KL-6 (U/ml)	673 [453-1,030] [b]
	CRP (mg/dl)	0.57 [0.15-1.78] [b]
	Lymphocyte (/µl)	971 [696-1,386] [b]
	Albumin (g/dl)	3.42 ± 0.56 [a]
	$PaCO_2$ (mmHg)	37.3 [34.3-40.3] [b]
	Ferritin (ng/ml)	360 [165-843] [b]
	IgG (mg/dl)	1,476 [1,295-1,813] [b]
Autoantibody *n* (%)	Anti-Jo-1 Ab	21/ 114 (18.4%)
	Anti-ARS Ab	9/ 45 (20.0%)
	Anti-MDA5 Ab	8/31 (25.8%)
	Anti-TIF-1γ Ab	2/2 (100%)
	ANA (>80×)	33/111 (29.7%)
	Anti-SS-A Ab	15/80 (18.8%)
Malignancy (<3 years) *n* (%)		21/112 (18.8%)
Treatment	Initial PSL dose (mg/kg/day)	0.83 ± 0.29 [a]
	mPSL pulse *n* (%)	77 /116 (66.4%)
	IVCY *n* (%)	48 /116 (41.4%)
	Calcineurin inhibitor *n* (%)	81/115 (70.4%)
	Combination therapy[c] *n* (%)	40 /116 (34.5%)
	IVIg *n* (%)	13/115 (11.3%)
Prognosis	Death *n* (%)	28 /116 (24.1%)
	ICU management *n* (%)	13 /116 (11.2%)
	Serious infection *n* (%)	38 /116 (32.8%)

[a] The data are shown as the mean ± standard deviation.

[b] Values are the median [interquartile range].

[c] Combination therapy includes glucocorticoid, IVCY and calcineurin inhibitors.
