# Supplementary material for: The predictive prognostic factors for polymyositis/dermatomyositis-associated interstitial lung disease
Source: Arthritis Res Ther. 2018 Jan 11;20:7. doi: 10.1186/s13075-017-1506-7 (PMC5765702; doi:10.1186/s13075-017-1506-7)
Supplement: Supplementary file 2 — Comparison of demographic data among each PM/DM subtype in PM/DM-ILD patients. (PDF 102 kb) [file 13075_2017_1506_MOESM2_ESM.pdf]

Table S2 Comparison of demographic data among each PM/DM subtype in PM/DM-ILD patients.

**PM/DM-ILD (*n* = 116)**		**PM (*n* = 22)**	**DM (*n* = 51)**	**CADM (*n* = 43)**	***p*-Value**
Women *n* (%)		13 /22 (59.1%)	40 /51 (78.4%)	30 /43 (69.8%)	0.23
Age (year)		56.2 ± 14.9 [a]	60.0 ± 13.3 [a]	51.3 ± 15.4 [a]	0.016**
Smoking *n* (%)		10 /21 (47.6%)	13 /47 (27.7%)	14 /38 (36.8%)	0.27
Follow-up period (months)		57.9 [48.4-78.7] [b]	42.4 [18.8-69.0] [b]	39.5 [14.5-80.0] [b]	0.081
Baseline data	CK (U/l)	1,956 [1,067-4,399] [b]	609 [211-1,773] [b]	83 [57-183] [b]	<0.001**
	LDH (U/l)	449 [374-564] [b]	437 [341-590] [b]	284 [242-343] [b]	<0.001**
	KL-6 (U/ml)	685 [490-1,709] [b]	759 [463-1,129] [b]	657 [413-911] [b]	0.35
	CRP (mg/dl)	0.71 [0.18-1.95] [b]	0.62 [0.16-1.94] [b]	0.49 [0.15-1.32] [b]	0.44
	Lymphocyte (/μl)	1,346 [983-1,553] [b]	971 [696-1,386] [b]	825 [592-1,041] [b]	0.022*
	Albumin (g/dl)	3.42 ± 0.49 [a]	3.32 ± 0.61 [a]	3.55 ± 0.51 [a]	0.16
	$PaCO_2$ (mmHg)	39.3 [38.2-41.8] [b]	37.0 [34.0-39.5] [b]	36.4 [34.1-39.9] [b]	0.14
	Ferritin (ng/ml)	258 [165-305] [b]	960 [719-1,365] [b]	361 [116-645] [b]	0.032*
	IgG (mg/dl)	1,580 [1,341-2,285] [b]	1,444 [1,221-1,731] [b]	1,489 [1,358-1,811] [b]	0.44
Autoantibody *n* (%)	Anti-Jo-1 Ab	8 /21 (38.1%)	10 /51 (19.6%)	3 /42 (7.1%)	0.011*
	Anti-ARS Ab	1 /2 (50.0%)	3 /21 (14.3%)	5 /22 (22.7%)	NA[d]
	Anti-MDA5 Ab	0 /1 (0%)	4 /17 (23.5%)	4 /13 (30.8%)	NA[d]
	Anti-TIF-1γ Ab	0 /0 (0%)	2 /2 (100%)	0 /0 (100%)	NA[d]
	ANA (>80×)	3 /20 (15.0%)	22 /48 (45.8%)	8 /43 (18.6%)	0.005**
	Anti-SS-A Ab	3 /14 (21.4%)	8 /36 (22.2%)	4 /30 (13.3%)	0.63
Malignancy (<3 years) *n (%)*		3 /20 (15.0%)	12 /49 (24.5%)	6 /43 (14.0%)	0.39
HRCT	Zone A	1.0 [0-1.0] [b]	1.0 [0-1.0] [b]	1.0 [0-1.0] [b]	0.90
	Zone B	1.0 [1.0-1.8] [b]	1.0 [0-2.0] [b]	1.0 [0-1.0] [b]	0.58
	Zone C	2.0 [1.3-3.0] [b]	1.0 [1.0-3.0] [b]	1.0 [1.0-2.0] [b]	0.22
	Zone D	4.0 [3.0-4.8] [b]	3.0 [2.0-4.0] [b]	3.0 [2.0-3.0] [b]	0.033*
	Zone total	8.0 [5.3-10.8] [b]	6.0 [3.0-10.0] [b]	6.0 [4.0-7.0] [b]	0.25
Treatment	Initial PSL dose (mg/kg/day)	0.77 ± 0.25 [a]	0.92 ± 0.28 [a]	0.72 ± 0.32 [a]	0.004**
	mPSL pulse *n* (%)	10 /22 (45.5%)	35 /51 (68.6%)	32 /43 (74.4%)	0.059
	IVCY *n* (%)	6 /22 (27.3%)	19 /51 (37.3%)	23 /43 (53.5%)	0.092
	Calcineurin inhibitor *n* (%)	8 /22 (36.4%)	36 /50 (72.0%)	37 /43 (86.0%)	<0.001**
	Combination therapy[c] *n* (%)	2 /22 (9.1%)	18 /51 (35.3%)	20 /43 (46.5%)	0.011*
	IVIg *n* (%)	1 /22 (4.5%)	10 /50 (20.0%)	2 /43 (4.7%)	NA[d]
Prognosis	Death *n* (%)	6 /22 (27.3%)	15 /51 (29.4%)	7 /43 (9.6%)	0.31
	ICU management *n* (%)	2 /22 (9.1%)	7 /51 (13.7%)	4 /43 (9.3%)	NA[d]

[a] The data are shown as the mean ± standard deviation.

[b] Values are the median [interquartile range].

[c] Combination therapy includes glucocorticoid, IVCY and calcineurin inhibitors.

[d] Not applicable.

**p* < 0.05, ***p* < 0.01.
