# Supplementary material for: The predictive prognostic factors for polymyositis/dermatomyositis-associated interstitial lung disease
Source: Arthritis Res Ther. 2018 Jan 11;20:7. doi: 10.1186/s13075-017-1506-7 (PMC5765702; doi:10.1186/s13075-017-1506-7)
Supplement: Supplementary file 3 — Comparison of demographic data between survivors and non-survivors of PM/DM-ILD patients. (PDF 92 kb) [file 13075_2017_1506_MOESM3_ESM.pdf]

Table S3 Comparison of demographic data between survivors and non-survivors of PM/DM-ILD patients.

**PM/DM-ILD (*n* = 116)**		**Non-survivor [e] (*n* = 14)**	**Survivor (*n* = 102)**	***p*-Value**
Women *n* (%)		8 /14 (57.1%)	75 /102 (73.5%)	0.22
Type (*n*)		PM 1, DM 8, CADM 5	PM 21, DM 43, CADM 38	0.41
Age (year)		60.1 ± 9.6 [a]	55.5 ± 15.4 [a]	0.14
Smoking *n* (%)		4/11 (36.4%)	33 /95 (34.7%)	1.00
Follow-up period (months)		1.65 [1.3-2.15] [b]	50.0 [32.7-79.6] [b]	<0.001**
Manifestation *n* (%)	Eruption	13 /14 (92.9%)	84/101 (83.2%)	0.69
	Muscle weakness	9 /14 (64.3%)	67 /101 (66.3%)	1.00
	Dysfunction of swallowing	3 /14 (21.4%)	19 /100 (19.0%)	0.73
	Fever	9 /14 (64.3%)	44 /101 (43.6%)	0.15
aseline data	CK (U/l)	377 [118-1,083] [b]	361 [118-1,691] [b]	0.79
	LDH (U/l)	438 [337-538] [b]	364 [276-516] [b]	0.21
	KL-6 (U/ml)	935 [809-1,230] [b]	612 [431-1,023] [b]	0.016*
	CRP (mg/dl)	1.32 [0.58-1.96] [b]	0.49 [0.12-1.62] [b]	0.072
	Lymphocyte (/μl)	714 [398-909] [b]	1,013 [730-1,425] [b]	0.002**
	Albumin (g/dl)	3.03 ± 0.54 [a]	3.46 ± 0.55 [a]	0.014*
	$PaCO_2$ (mmHg)	32.6 [31.2-36.0] [b]	37.8 [35.3-40.7] [b]	0.005**
	Ferritin (ng/ml)	658 [148-1,210] [b]	342 [165-734] [b]	0.45
	IgG (mg/dl)	1,714 [1,436-1,876] [b]	1,475 [1,287-1,784] [b]	0.39
Autoantibody *n* (%)	Anti-Jo-1 Ab	1/ 14 (7.1%)	20 /100 (20.0%)	0.46
	Anti-ARS Ab	0 /3 (0%)	9 /42 (21.4%)	1.00
	Anti-MDA5 Ab	-	8 /31 (25.8%)	NA [f]
	Anti-TIF-1γ Ab	-	2 /2 (100%)	NA [f]
	ANA (>80×)	4 /12 (33.3%)	29 /99 (29.3%)	0.75
	Anti-SS-A Ab	0 /7 (0%)	15 /73 (20.5%)	0.34
Malignancy (<3 years) *n* (%)		3 /12 (25.0%)	18 /100 (18.0%)	0.69
HRCT	Zone A	2.0 [1.0-2.0] [c]	1.0 [0-1.0] [c]	< 0.001**
	Zone B	2.0 [1.0-2.8] [c]	1.0 [0-1.0] [c]	0.001**
	Zone C	2.0 [1.0-3.8] [c]	1.0 [1.0-2.0] [c]	0.069
	Zone D	4.0 [3.0-4.8] [c]	3.0 [2.0-4.0] [c]	0.021*
	Zone total	10.0 [6.5-13.5] [c]	6.0 [4.0-8.3] [c]	0.004**
Treatment	Initial PSL dose (mg/kg/day)	0.96 ± 0.24 [a]	0.80 ± 0.31 [a]	0.061
	mPSL pulse *n* (%)	14 /14 (100%)	63 /102 (61.8%)	0.002**
	IVCY *n* (%)	11 /14 (78.6%)	37 /102 (36.3%)	0.003**
	Calcineurin inhibitor *n* (%)	13 /14 (92.9%)	68 /101 (67.3%)	0.062
	Combination therapy[c] *n* (%)	10 /14 (71.4%)	30 /102 (29.4%)	0.005**
	IVIg *n* (%)	4 /14 (28.6%)	9 /101 (8.9%)	0.052

	ICU management *n* (%)	9 /14 (64.3%)	4 /102 (3.9%)	<0.001**
Outcome	Serious infection *n* (%)	11 /14 (78.6%)	27 /102 (26.5%)	<0.001**
	ST[d] *n* (%)	8 /14 (57.1%)	63 /96 (65.6%)	0.56
	β-D-glucan *n* (%)	6 /13 (46.2%)	8 /95 (8.4%)	0.002**
	CMV treatment *n* (%)	5 /13 (38.5%)	18 /93 (19.4%)	0.15

[a] The data are shown as the mean ± standard deviation.

[b] Values are the median [interquartile range].

[c] Combination therapy includes glucocorticoid, IVCY and calcineurin inhibitors.

[d] ST stands for sulfamethoxazole/trimethoprim.

[e] The non-survivor group includes the patients who die within 6 months after the diagnosis of PM/DM.

[f] Not applicable.

*$p < 0.05$, **$p < 0.01$.
