# Supplementary material for: The predictive prognostic factors for polymyositis/dermatomyositis-associated interstitial lung disease
Source: Arthritis Res Ther. 2018 Jan 11;20:7. doi: 10.1186/s13075-017-1506-7 (PMC5765702; doi:10.1186/s13075-017-1506-7)
Supplement: Supplementary file 4 — Comparison of demographic data between PM/DM-ILD patients with or without complication of serious infection. (PDF 92 kb) [file 13075_2017_1506_MOESM4_ESM.pdf]

Table S4 Comparison of demographic data between PM/DM-ILD patients with or without complication of serious infection[a].

**PM/DM-ILD (*n* = 116)**			**Infection (*n* = 38)**	**Non-infection (*n* = 78)**	***p*-Value**
Women *n* (%)			24 /38 (63.2%)	59 /78 (75.6%)	0.16
Type (*n*)			PM 7, DM 24, CADM 7	PM 15, DM 27, CADM 36	0.007**
Age (year)			58.9 ± 13.9 [b]	54.7 ± 15.2 [b]	0.15
Smoking *n* (%)			10 /35 (28.6%)	27 /71 (38.0%)	0.34
Follow-up period (months)			32.1 [2.8-62.1] [c]	49.1 [31.6-79.6] [c]	0.024*
Manifestation	Eruption		32 /38 (84.2%)	65 /77 (84.4%)	0.98
n (%)	Muscle weakness		28 /38 (73.7%)	48 /77 (62.3%)	0.23
	Dysfunction of swallowing		9 /38 (23.7%)	13 / 76 (17.1%)	0.40
	Fever		21 /38 (55.3%)	32 / 77 (41.6%)	0.17
Baseline data	CK (U/l)		418 [191-1,136] [c]	220 [76-1,887] [c]	0.40
	LDH (U/l)		420 [335-574] [c]	343 [258-486] [c]	0.005**
	KL-6 (U/ml)		848 [605-1,231] [c]	572 [405-978] [c]	0.014*
	CRP (mg/dl)		0.9 [0.34-1.99] [c]	0.30 [0.10-1.51] [c]	0.032*
	Lymphocyte (/μl)		848 [552-1,177] [c]	1,008 [735-1,435] [c]	0.040*
	Albumin (g/dl)		3.18 ± 0.52 [b]	3.53 ± 0.55 [b]	0.003**
	$PaCO_2$ (mmHg)		36.0 [31.5-38.8] [c]	38.0 [35.5-41.5] [c]	0.016*
	Ferritin (ng/ml)		462 [223-1,369] [c]	313 [142-666] [c]	0.071
	IgG (mg/dl)		1,420 [1,208-1,714] [c]	1,475 [1,287-1,784] [c]	0.12
Autoantibody	Anti-Jo-1 Ab		7 /38 (18.4%)	14 /76 (18.4%)	1.00
n (%)	Anti-ARS Ab		4 /17 (23.5%)	5 /28 (17.9%)	0.71
	Anti-MDA5 Ab		4 /15 (26.7%)	4 /16 (25.0%)	1.00
	Anti-TIF-1γ Ab		-	2 /2 (100%)	NA[e]
	ANA (>80×)		10 /35 (28.6%)	23 /76 (30.3%)	0.86
	Anti-SS-A Ab		3 /23 (13.0%)	12 /57 (21.1%)	0.54
Malignancy (<3 years) *n (%)*			8 /36 (22.2%)	13 /76 (17.1%)	0.52
HRCT	Zone A		1.0 [0-2.0] [c]	1.0 [0-1.0] [c]	0.054
	Zone B		1.0 [1.0-2.0] [c]	1.0 [0-1.0] [c]	0.049*
	Zone C		2.0 [1.0-3.0] [c]	1.0 [1.0-2.0] [c]	0.036*
	Zone D		3.0 [2.0-4.0] [c]	3.0 [2.0-4.0] [c]	0.068
	Zone total		7.0 [4.0-11.0] [c]	6.0 [4.0-8.0] [c]	0.031*
Treatment	Initial PSL dose (mg/kg/day)		0.94 ± 0.30 [b]	0.76 ± 0.29 [b]	0.005**
	Accumulative	1 month	23.7 ± 7.5 [b]	21.5 ± 7.7 [b]	0.38
	PSL dose	2 months	41.4 ± 15.2 [b]	38.7 ± 13.3 [b]	0.62
	(mg/kg)	4 months	64.2 ± 28.0 [b]	64.0 ± 20.9 [b]	0.61
		6 months	90.0 ± 31.9 [b]	82.8 ± 27.2 [b]	0.60
	mPSL pulse *n* (%)		31 /38 (81.6%)	46 /78 (59.0%)	0.016*
	IVCY *n* (%)		25 /38 (65.8%)	23 /78 (29.5%)	<0.001**

	Calcineurin inhibitor *n* (%)	34 /38 (89.5%)	47 /77 (61.0%)	0.002**
	Combination therapy [d] *n* (%)	23 /38 (60.5%)	17 /78 (21.8%)	<0.001**
	IVIg *n* (%)	6 /38 (15.8%)	7 /77 (9.1%)	0.35
Outcome	ICU management *n* (%)	10 /38 (26.3%)	3 /78 (3.8%)	0.001**
	Death *n* (%)	11 /38 (28.9%)	3 /78 (3.8%)	<0.001**

[a] The infection group includes the PM/DM-ILD patients who have the complication of serious infections which need intravenous antibiotic therapy or longer hospitalization within 6 months after the diagnosis.

[b]The data are shown as the mean ± standard deviation

[c]Values are the median [interquartile range]

[d]Combination therapy including mPSL, IVCY, and calcineurin inhibitor

[e]Not applicable

*$p < 0.05$, **$p < 0.01$
